# A Misunderstood Scientist

**Published:** 1907-06

**Authors:** 


### A MISUNDERSTOOD SCIENTIST.

Dr. William Osler's statement made nearly two years ago, that "man's best work is done before 40," which he meant in a preparatory sense, created a world-wide sensation at the time, its signification being thoughtlessly and injuriously perverted. It is only lately that it has received its true interpretation.

Taking advantage of Dr. Osler's presence in Canada recently (whither he came to celebrate the centenary of his beloved mother,) Mr. Charles Leonard-Stuart, the well-known encyclopedist, wrote to him for accurate information. The reports of the professor's famous address to the students at Johns Hopkins University were so distorted that "oslerized" has become a familiar term.

Dr. Osler's real words on that occasion were:

"The teacher's life should have three periods—study until 25, investigation until 40, profession until 60, at which time I would have him retired on a double allowance. Whether Anthony Trolope's suggestion of a college and chloroform should be carried out or not, I have become a little dubious, as my own time is getting so short."

This is very different from the generally-accepted idea that he asserted that man's creative usefulness ended at 40, and suggested his chloroforming at 60. In an encyclopedic article on Medicine, Dr. Osler writes thus on age:

"Within the past three centuries the average working life of English-speaking men has doubled. A few lived as long as now, and some strong or favored ones had efficient working powers as long; but the common life was worn out in what is now middle age. In Shakespeare's time the 50s were venerable; 'Old John of Gaunt, time-honored Lancaster,' was 58 when supposably so addressed; and Admiral Coligny, murdered at 53, is described by his contemporary biographer as a very old man. Now when we hear of a death in the 60s we instinctively feel it an untimely cutting-off in what should be still fresh and vigorous age, and even at 80 it seems but just fair ripeness for the sickle."

Even at this late date it is a matter of general interest to have the professor's position accurately defined. It was a singular instance of a man suddenly becoming famous for what he did not say, whereas what he did say was very much more to the point than the sentiments popularly but erroneously attributed to him.—Christian Herald.

